# fMRI neurofeedback facilitates anxiety regulation in females with spider phobia

**DOI:** 10.3389/fnbeh.2015.00148

**Published:** 2015-06-08

**Authors:** Anna Zilverstand, Bettina Sorger, Pegah Sarkheil, Rainer Goebel

**Affiliations:** ^1^Department of Cognitive Neuroscience, Maastricht UniversityMaastricht, Netherlands; ^2^Department of Psychiatry, Icahn School of Medicine at Mount SinaiNew York, NY, USA; ^3^Department of Psychiatry, Psychotherapy and Psychosomatics, RWTH Aachen University HospitalAachen, Germany; ^4^Department of Neuroimaging and Neuromodeling, Netherlands Institute for NeuroscienceAmsterdam, Netherlands

**Keywords:** fMRI, real-time, self-regulation, neurofeedback, spider phobia, anxiety, regulation, cognitive reappraisal

## Abstract

**Background:** Spider phobics show an exaggerated fear response when encountering spiders. This fear response is aggravated by negative and irrational beliefs about the feared object. Cognitive reappraisal can target these beliefs, and therefore has a fear regulating effect. The presented study investigated if neurofeedback derived from functional magnetic resonance imaging (fMRI) would facilitate anxiety regulation by cognitive reappraisal, using spider phobia as a model of anxiety disorders. Feedback was provided based on activation in left dorsolateral prefrontal cortex and right insula, as indicators of engagement and regulation success, respectively.

**Methods:** Eighteen female spider phobics participated in a randomized, controlled, single-blinded study. All participants completed a training session in the MRI scanner. Participants assigned to the neurofeedback condition were instructed to shape their regulatory strategy based on the provided feedback. Participants assigned to the control condition were asked to adapt their strategy intuitively.

**Results:** Neurofeedback participants exhibited lower anxiety levels than the control group at the end of the training. In addition, only neurofeedback participants achieved down-regulation of insula activation levels by cognitive reappraisal. Group differences became more pronounced over time, supporting learning as a mechanism behind this effect. Importantly, within the neurofeedback group, achieved changes in insula activation levels during training predicted long-term anxiety reduction.

**Conclusions:** The conducted study provides first evidence that fMRI neurofeedback has a facilitating effect on anxiety regulation in spider phobia.

## Introduction

Interest in novel treatment approaches for patients with anxiety disorders is high. Anxiety disorders are the most common mental health condition, with a year-prevalence of 12–18% (Wittchen and Jacobi, 2005; Kessler et al., 2011, 2012). Moreover, 16–47% of these patients cannot be treated successfully with a currently standard treatment such as cognitive behavior therapy (Ost, 2008). Further integration of cognitive regulation strategies into the treatment of anxiety disorders has therefore been suggested (Kamphuis and Telch, 2000; Amstadter, 2008; Farmer and Kashdan, 2012). ”Cognitive reappraisal,” the reinterpretation of the meaning of a stimulus, is an effective emotion regulation strategy, with beneficial long-term impact on anxiety symptoms (Kamphuis and Telch, 2000; Sloan and Telch, 2002; Amstadter, 2008; Farmer and Kashdan, 2012). This method targets negative, anxiety-provoking beliefs, which undermine regulation and prevent an adaptive response to the perceived threat (Gross, 1998; Amstadter, 2008). Spider phobics, similar to patients with other anxiety disorders, hold these beliefs (Arntz et al., 1993), and are expected to benefit from training reappraisal. The aim of this study was to investigate if providing neurofeedback during cognitive reappraisal would facilitate regulation success in spider phobia as a specific form of anxiety disorders.

Neurofeedback training based on functional magnetic resonance imaging (fMRI) is increasingly gaining interest as a novel approach in treating neurological and psychiatric disorders. This method suggests that the presentation of feedback derived from patients' current neural activation can train the voluntarily regulation of selected brain processes. The goal is to achieve a normalization of deviant brain processes, and thus improve the related behavioral symptoms. Previous studies found that neurofeedback is an efficient tool in shaping mental strategies toward a given goal (DeCharms et al., 2005; Caria et al., 2007; Linden et al., 2012; Scheinost et al., 2013; Young et al., 2014). Exploratory investigations have also indicated a benefit of fMRI neurofeedback training in clinical populations with chronic pain, tinnitus, Parkinson's disease, stroke, and mood disorders (DeCharms et al., 2005; Haller et al., 2010; Subramanian et al., 2011; Linden et al., 2012; Sitaram et al., 2012; Young et al., 2014). Neurofeedback training methods have not been applied in patients with anxiety disorders, but it has been demonstrated that subclinical levels of anxiety can be successfully reduced by learning self-regulation of select brain activation levels (Scheinost et al., 2013).

Spider phobia, as other anxiety disorders, is characterized by an exaggerated fear response when encountering the feared object, in this case spiders. This strong fear response is accompanied by hyperactivation of a network of brain regions involved in anxiety expression (the anxiety expression network), such as the amygdala and insula (Etkin and Wager, 2007). Both amygdala and insula have been proposed to belong to a core anxiety network implicated across different anxiety disorders (Etkin and Wager, 2007). While the amygdala has been linked to initial automatic fear processing during fear expression (Carlsson et al., 2004; Straube et al., 2006), the insula represents sustained anxious emotion (Somerville et al., 2013). Successful fear regulation in healthy subjects is characterized by down-regulation of this anxiety expression network (Delgado et al., 2008), and reduced activation levels in this network have been linked to a positive treatment response (Schienle et al., 2007). Beyond the anxiety network, a reduced regulatory capacity during anxiety provocation was shown in spider phobics, marked by hypoactivation of a frontal regulatory network (New et al., 2009; Manber-Ball et al., 2013). This frontal network encompasses cingulate and prefrontal cortices, such as dorsolateral prefrontal cortex (dlPFC), and is known to be activated during the regulation of negative affect in healthy participants (Ochsner et al., 2012). Engagement of the dlPFC during cognitive reappraisal is delayed in patients with anxiety disorders, with the delay predicting levels of anxiety (Goldin et al., 2009). Furthermore, dlPFC activation levels during fear regulation are inversely associated with the severity of anxiety and functional impairment (New et al., 2009; Manber-Ball et al., 2013). Also, an increase in dlPFC activation levels predicts treatment success (Hauner et al., 2012). In healthy participants, dlPFC has been implicated in safety learning and successful anxiety regulation (Delgado et al., 2008; Pollak et al., 2010).

The neurofeedback training implemented in this study provided patients with a novel dual feedback display. Participants received feedback on both their current activation levels of the insula (sustained anxious emotion) and the dlPFC (engagement in regulation) during anxiety regulation. Neurofeedback participants were asked to continuously improve their regulation strategy according to the feedback, while a non-feedback control group was asked to learn based on intuition. We expected reduced insula activation in combination with high dlPFC activation in the neurofeedback group in comparison to the control group. Additionally, we hypothesized that this normalization of brain activation patterns in the neurofeedback group would predict reduced immediate- and long-term subjective levels of spider fear. A link between successful self-regulation of brain activation levels and long term behavioral change would provide first evidence that neurofeedback may be an efficacious tool for enhancing anxiety regulation.

## Materials and methods

### Participants

Eighteen women were recruited through public advertisement at Maastricht University. They were screened for high spider fear [Spider Phobia Questionnaire (SPQ) Score ≥ 14, (Klorman et al., 1974)] and diagnosed with spider phobia according to the criteria of The Diagnostic and Statistical Manual of Mental Disorders DSM-IV TR (American Psychiatric Association, 2000). All were free of psychotropic medication and were not affected by other current or previous neuropsychiatric comorbidities as evaluated by means of a structured clinical interview [Mini International Neuropsychiatric Interview, MINI, (Sheehan et al., 1998)]. None of the participants had previously received cognitive behavioral therapy. All participants were students, or currently employed. To balance the two experimental groups for age, self-reported use of reappraisal strategies [Emotion Regulation Questionnaire, Reappraisal score, ERQ-R, (Gross and John, 2003)], and spider fear (SPQ score), we used a restricted randomization procedure shown to be efficient for small sample sizes [sequential balancing, (Borm et al., 2005)] (Table 1). Participants were naïve to group assignment and goal of the study. They were informed that they were participating in a treatment study investigating a novel anxiety regulation technique. All participants were equally compensated (8 €/h) and gave their written informed consent prior to the experiment according to the Declaration of Helsinki and approved by the local Medical Ethics Committee at Maastricht University.

**Table 1:** **Characteristics of study participants**.

**Variables (mean ± SD)**	**Control group**	**Neurofeedback group**	***p*-value**
Gender (female)	*n* = 9	*n* = 9	
Age	21.7 (2.1)	20.7 (1.2)	0.23
Duration (years)	15.0 (3.0)	14.0 (1.9)	0.42
ERQ-R	30.1 (3.5)	29.1 (2.8)	0.52
SPQ	19.2 (2.9)	19.3 (3.4)	0.94
FSQ	90.0 (14.8)	91.2 (10.9)	0.84
SBQ	56.2 (10.5)	54.9 (7.9)	0.77

*Duration, since onset of symptoms; ERQ-R, Emotion Regulation Questionnaire Reappraisal Score; SPQ, Spider Phobia Questionnaire; FSQ, Fear of Spider questionnaire; SBQ, Spider Belief Questionnaire*.

### Procedure

Participants first had a 15-min practice session on how to use cognitive reappraisal during provocation of anxiety by spider photographs. An instructor (clinical psychologist) guided the participants to reinterpret a situation by “finding out calming aspects” instead of “engaging in anxiety provoking thoughts.” Participants were told that the rationale was to normalize some of the most common negative beliefs held by spider phobics (Arntz et al., 1993), drawing the focus to the safety of the situation. They were asked to select from four sorts of strategies: (1) detecting the aesthetics of the spider, (2) focusing on its powerlessness, (3) changing its connotation by humanizing it, or (4) changing its context by imagining approaching it in a safe environment. Each participant was invited to write down their own personal credible version of each strategy. They were then familiarized with the MRI procedures and requested to rehearse aloud during eight practice trials *(regulate trials*). Last, participants were asked to practice refraining from changing their thoughts in another eight practice trials, letting thoughts occur spontaneously (*watch trials*).

Neurofeedback participants were introduced to the dual feedback display and explained the feedback rationale. They were instructed to adjust the reappraisal strategy based on the provided feedback throughout the experiment. They were told that the goal was to achieve high prefrontal activation (“reappraisal activation”) and reduced insular activation (“anxiety activation”). Participants were asked to primarily consider the feedback from the regulatory network, if dual feedback information was challenging. The control group was presented a visually similar display, and was instructed that it indicated a short break in-between trials. Control participants were asked to adapt their strategy based on intuition throughout the session. All participants were told that experiencing high anxiety levels may be an essential part of the regulation process, and is generally not harmful. They were reminded that they could stop at any time, asked to pay attention, and to refrain from any movements in the scanner. Immediately before the imaging session, all participants completed the Questionnaire of Current Motivation [QCM, (Rheinberg et al., 2001)], which measures individual differences in current motivation and expectation of success.

**Figure 1:**
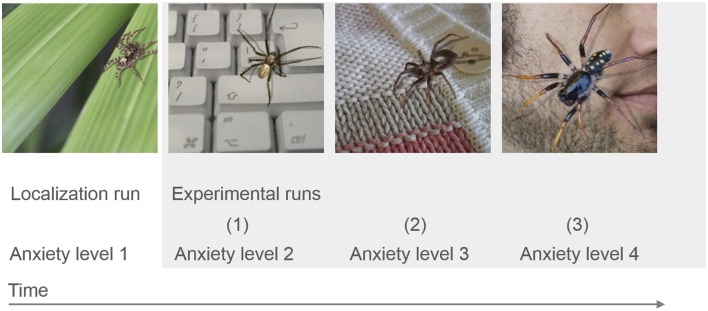
**fMRI study design**. The stimuli used were selected to be increasingly anxiety provoking with each run, based on a behavioral pilot study (Supplementary Figure 1). Participants from the neurofeedback group received feedback during the three experimental runs, after the individual target regions had been defined based on the localization run.

**Figure 2:**
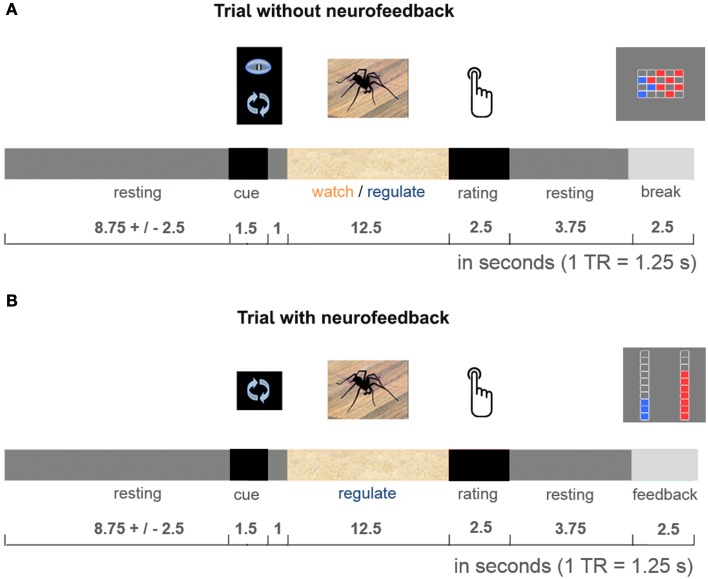
**Cognitive reappraisal trial**. Participants were either asked to let their thoughts occur spontaneously (*watch trials:* identical in both groups, **A**), or were cued to use cognitive reappraisal (*regulate trials:* neurofeedback provided during experimental runs in neurofeedback group, **B**; break display presented in control group, **A**). They performed the task with these respective instructions during the 12.5-s stimulus presentation. After each active trial period participants rated their subjective anxiety. Then the dual feedback display, or “break display” was presented.

The 50-min imaging session started with one 5-min anatomical imaging run, followed by four 11-min functional imaging runs. Participants performed the practiced task during all four functional runs, alternating *regulate* and *watch* trials (presented in a blocked design, e.g., 4 *watch trials*, 4 *regulate trials*, 4 *watch trials*, 4 *regulate trials*, counterbalanced order). Data from the first functional run were used for delineation of the dlPFC and insula target regions (localization run). Neurofeedback was presented from the second to fourth functional run (neurofeedback training). To keep the training challenging throughout the experiment, the presented stimuli were selected to be increasingly anxiety provoking with each run (Figure 1). All stimuli were selected based on a behavioral pilot study with spider phobics (Supplementary Figure 1), and presented only once per condition. Each trial started with a 1.5-s cue (pictogram: *watch* or *regulate*), followed by 1-s fixation and the 12.5-s active trial period of anxiety regulation during presentation of the spider photograph (Figure 2). Participants then rated their subjective anxiety on a 5-point Likert scale from 0 = “not fearful at all” to 4 = “extremely fearful” using a button box (Current Designs, Philadelphia, PA, USA). The feedback display was presented to the neurofeedback group after regulate trials, 2.5 s after the trials elapsed. During watch trials, and in the control group the “break display” was shown. All displays were presented using Presentation (Version 16; Neurobehavioral Systems, Albany, USA). Between trials there was a jittered resting period of 8.75 ± 2.5-s. We chose to present intermittent feedback to avoid cognitive overload and distraction, improve signal to noise ratio of the feedback signal, and accommodate hemodynamic delay (Stoeckel et al., 2014). Intermittent feedback paradigms have been empirically demonstrated to be effective in shaping neural activity and learning (Bray et al., 2007; Johnson et al., 2012).

After the training session participants were asked to indicate which reappraisal strategy they believed to be the most successful one (“which strategy would you recommend?”), to rate on a 7-point Likert scales how helpful the reappraisal instruction (both groups) and the provided neurofeedback (only experimental group) were, if neurofeedback was helpful in selecting a reappraisal strategy (only experimental group), how comfortable they were in the scanner environment (both groups), and to indicate their willingness to come back for another session (both groups).

### MRI imaging

Images were acquired at Maastricht Brain Imaging Centre (Maastricht University) on a 3T scanner (Tim Trio/upgraded to Prisma Fit, Siemens Healthcare, Germany). The functional echo-planar imaging (EPI) sequence was optimized for imaging of limbic and prefrontal regions (Weiskopf et al., 2007; Morawetz et al., 2008): repetition time = 1250 ms, echo time = 25 ms, flip angle = 67°, slice thickness = 2.5 mm, 20% gap, in-plane = 3 × 3 mm, slice angle of 25–30°, grappa acceleration = 2. We compromised for coverage of parietal cortex to achieve higher sampling rate for real-time imaging analysis. Heart and breathing rates were monitored using Siemens pulse oximeter and breathing chest band (recording the first 5 min of each 11-min functional run). Anatomical images were collected with a magnetization-prepared rapid acquisition gradient echo (3D MPRAGE) sequence: repetition time = 1900 ms, echo time = 2.52 ms, flip angle = 9°, voxel size 1 × 1 × 1 mm^3^, with duration 4:26 min.

### Real-time imaging analysis

**Figure 3:**
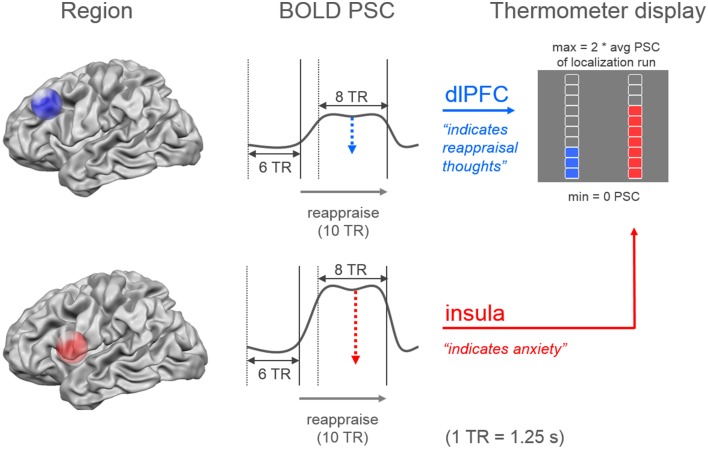
**Neurofeedback calculation**. The dual neurofeedback display showed current individual activation level in dorsolateral prefrontal cortex (blue), and insula (red). Neurofeedback participants were instructed that the blue thermometer reflected their engagement in reappraisal thoughts, while the red thermometer indicated their anxiety level. Each thermometer was individually adjusted according to average activation level during localization run (avg PSC = average percent signal change). Neurofeedback values were based on the increase in percent signal change during the last 10-s (8 TR = time to repetition) of stimulus presentation, relative to a 7.5-s period (6 TR) previous to stimulus onset.

During the imaging session all functional images were analyzed with Turbo-BrainVoyager (Version 3.0; Brain Innovation, Maastricht, Netherlands). The images were pre-processed using motion correction, drift confound predictors, and high-pass filtering with a general linear model (GLM) Fourier basis set (2 cycles). An incremental GLM was computed using two task predictors (*watch, regulate*) convolved by a standard two-gamma hemodynamic response function, as well as predictors for events of no interest. Functional maps were thresholded (*t* = 3, cluster threshold = 4 voxels). Target regions were individually defined based on the contrasts *watch* vs. resting (insula) and *regulate* vs. resting (dlPFC). The cluster closest to the target coordinates was manually selected. The target coordinates were defined unilaterally based on previous research: *x* = 37, *y* = 11, *z* = 3 in the right insula and *x* = −43, *y* = 28, *z* = 30 in the left dlPFC (Etkin and Wager, 2007; Delgado et al., 2008; Ochsner et al., 2012). During experimental runs, neurofeedback values were computed by contrasting the activation increase during stimulus presentation (last 10-s) relative to a baseline previous to stimulus onset (7.5-s) (Figure 3). The feedback was displayed on a thermometer, which had its maximum adjusted to average activation during the localization run (max thermometer = 2^*^ average activation localization run).

### *Post-hoc* imaging analysis

Functional and anatomical images were pre-processed *post-hoc* in BrainVoyager (Version QX 2.7; Brain Innovation, Maastricht, Netherlands) as during real-time analysis. None of the participants moved more than 3.0 mm/degrees in any direction/rotation. All data was spatially normalized to Talairach space to enable comparison between participants. Beta estimates for the modeled individual blood oxygen level-dependent (BOLD) response (*watch, regulate*) were derived for the individually defined target regions to perform a region-of-interest analysis. Separate analysis of the localization run and experimental runs were performed in SPSS Statistics (IBM 21; SPSS Statistics; IBM, Armonk, NY, USA). The beta weights were submitted to a repeated measures GLM with linear contrasts, modeling within factors *task (watch, regulate), functional run (1; 2–4), and group* as a between factor. Effect sizes were estimated using partial eta squared (Cohen, 1973).

For whole-brain random-effects GLM analysis the data was spatially smoothed (FWHM 6 mm) and noise confounds were added to represent the six head motion parameters (Weissenbacher et al., 2009), a localized estimate of white matter signal for modeling scanner artifacts (Jo et al., 2010), and the ventricular signal to control for physiological artifacts (Birn et al., 2009). Whole brain analyses statistical maps were thresholded with an initial uncorrected voxel-threshold of α = 0.05, and cluster-size threshold with a false positive rate of α = 0.05, (Forman et al., 1995).

### Physiological data

Pulse and breathing rate from each participant were computed per *task* condition (*resting, watch, regulate*) using a custom made MATLAB tool (R2010a; The Mathworks, Natick, USA). Physiological data were analyzed statistically as the imaging data.

### Behavioral data during training

Group differences during the training regarding motivation, expectation of success, comfort in the scanner, helpfulness of instruction and neurofeedback, and willingness to come back for additional sessions were statistically evaluated using independent sample *t*-tests. Subjective anxiety ratings collected during the imaging session were submitted to the same repeated measures GLM as the imaging data.

### Follow-up assessment spider fear

To evaluate long-term changes in spider fear the participants were followed during a period of 3 months. Spider fear was measured during screening, after the MRI training session, 2 weeks, and 3 months after the training. At each time point, participants were administered two questionnaires: the Fear of Spider Questionnaire [FSQ, (Szymanski, 1995)], selected for its high test-retest stability and internal consistency (Muris and Merckelbach, 1996), and the Spider Belief Questionnaire [SBQ, (Arntz et al., 1993)], which was specifically designed to measure the changes in beliefs held by spider phobics. Questionnaire data was analyzed using a repeated measures GLM with linear contrasts, within factor *time (screening, post-fMRI, 2-week, 3-month,)* and group as a between factor. To test for transfer from changes on a brain level to post-training behavioral change, we regressed change in spider fear (from *screening to 3-month*) on change in BOLD activation (from *localization run to last experimental run*) by simple linear regression.

## Results

### Behavioral data at baseline

Participants demonstrated similarly high levels of spider fear during screening (Table 1). Also, they had comparable levels of motivation and expectation of success prior to the training (Table 2).

**Table 2:** **Current Motivation and willingness to return**.

**Variables (mean ± SD)**	**Control group**	**Neurofeedback group**	***p*-value**
QCM fear	3.1 (1.1)	2.7 (1.1)	0.39
QCM challenge	5.8 (0.7)	5.7 (0.9)	0.77
QCM interest	5.4 (1.0)	5.3 (0.7)	0.79
QCM mastery	5.4 (0.5)	5.7 (0.7)	0.46
2nd session	5.9 (1.3)	6.0 (1.0)	0.84

*QCM, Questionnaire of Current Motivation (fear = incompetence fear, challenge = perceived challenge, interest = level of interest, mastery = mastery confidence); 2nd session, willingness to return (Likert scale 1–7)*.

### Localization run

**Figure 4:**
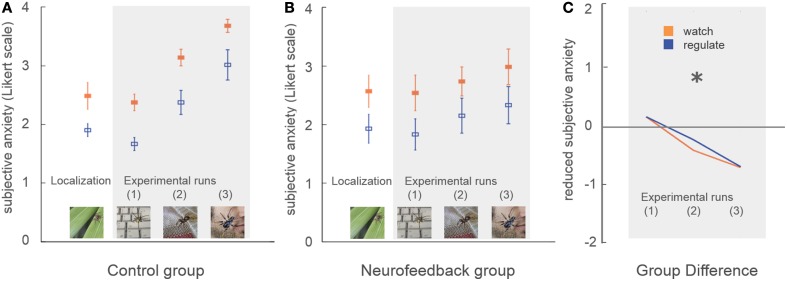
**Subjective anxiety**. Participants rated their subjective anxiety level on a five-point Likert scale from 0 = “not fearful at all” to 4 = “extremely fearful.” There was no baseline difference in average anxiety level, or in the ability to perform the task during the localization run **(A,B)**. During neurofeedback training (experimental runs) both groups achieved lower levels of anxiety during regulation trials (blue) in comparison to watch trials (orange). A marked group difference in general anxiety level emerged over time (marked with asterisk, **C**). While control participants demonstrated an increase of anxiety with increasingly challenging stimuli, this effect was attenuated in neurofeedback participants.

Subjective anxiety ratings demonstrated no group difference in average anxiety level at the beginning of the training (*p* = 0.84, Figure 4). Participants were comparable in ability to down-regulate anxiety during the initial localization run (*p* = 0.86, Figure 4).

**Figure 5:**
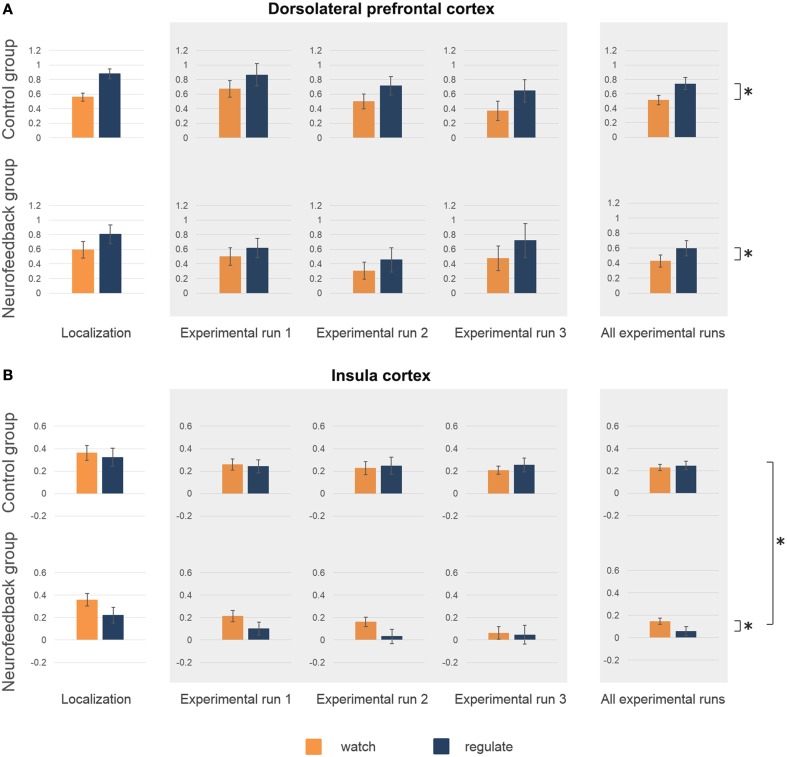
**Activation level target regions**. Activation level (percent signal change) in dorsolateral prefrontal cortex (dlPFC) **(A)** and insula **(B)** are depicted for the localization run, the experimental runs and averaged across experimental runs for both groups. There were no significant group differences in average activation levels, or in the ability to regulate during the localization run **(A,B)**. High activation levels in dlPFC during neurofeedback training (experimental runs) indicated high engagement of both groups **(A)**. Both group showed increased activation levels during *regulate* (blue) in comparison to *watch* (orange) trials (marked with asterisk, **A**). **(B)** A group difference in insula activation level emerged over time (marked with asterisk, **B**), with only neurofeedback participants showing a reduction of activation levels. Furthermore, only the neurofeedback group succeeded in achieving down-regulation of insula activation levels during experimental runs (marked with asterisk, **B**).

The average coordinates of the individually defined target regions were similar in both groups (max radial distance to intended target coordinates = 5 mm, Supplementary Figure 2, Supplementary Table 1). Average size of dlPFC and insula target regions were well matched (average size 12–15 functional voxels, Supplementary Table 1). Analysis of the right insula response showed no significant group difference for average activation (*p* = 0.58, Figure 5B), or ability to down-regulate insula activation levels during initial localization run (*p* = 0.11, Figure 5B). The response in left dlPFC indicated that both groups were highly engaged, as both achieved significant up-regulation of this region during *regulate* in comparison to *watch* trials [*F*_(1, 16)_ = 33.7, *p* < 0.001, η^2^_p_ = 0.68, Figure 5A]. There was no significant group difference regarding up-regulation (*p* = 0.35), or average dlPFC activation (*p* = 0.89).

### Neurofeedback training

Subjective anxiety ratings demonstrated that both groups were able to regulate anxiety to a certain extent, showing reduced anxiety during regulate trials [up-regulation: *F*_(1, 16)_ = 33.5, *p* < 0.001, η^2^*_p_* = 0.68]. Neurofeedback participants exhibited lower average anxiety levels than the control group, an effect which increased over time as stimuli became more challenging [time^*^group interaction: *F*_(1, 16)_ = 8.1, *p* < 0.05, η^2^*_p_* = 0.34]. While control participants demonstrated a marked increase in anxiety over time [*F*_(1, 8)_ = 33.3, *p* < 0.001, η^2^*_p_* = 0.81], this increase was attenuated in neurofeedback participants, who showed a non-significant trend [*F*_(1, 8)_ = 4.5, *p* = 0.07, Figure 4].

Analysis of the imaging data demonstrated that neurofeedback participants in comparison to the control group had significantly lower insula activation levels during regulate trials, but not during watch trials [group^*^condition interaction: *F*_(1, 16)_ = 7.8, *p* < 0.05, η^2^*_p_* = 0.33; regulate trials: *F*_(1, 16)_ = 7.7, *p* < 0.05, η^2^*_p_* = 0.33; watch trials: *F*_(1, 16)_ = 3.2, *p* = 0.09, Figure 5B]. *Post-hoc* within group analysis also demonstrated a significant reduction of insular activation levels over time in neurofeedback participants [*F*_(1, 8)_ = 7.1, *p* < 0.05, η^2^*_p_* = 0.47], but not in control participants (*p* = 0.33), as in the analysis of subjective ratings. Across participants, insula activation level (single trial betas) and subjective anxiety ratings (single trial ratings) were moderately correlated during both regulate and watch trials (both: *r* = 0.29, *p* < 0.01). Finally, analysis of insula activation levels revealed significantly better down-regulation during regulate trials in neurofeedback in comparison to control participants [*F*_(1, 16)_ = 7.8, *p* < 0.05, η^2^*_p_* = 0.33, Figure 5B]. *Post-hoc* within group tests demonstrated that the ability to down-regulate insula activation levels was significant in the neurofeedback group [*F*_(1, 8)_ = 6.7, *p* < 0.05, η^2^*_p_* = 0.46], but not the control group (*p* = 0.31). The whole-brain analysis further corroborated that neurofeedback participants achieved greater capacity for down-regulation within a network of brain regions involved in anxiety expression, including the right insula (Supplementary Table 2, Supplementary Figure 3). There was no significant group difference for average dlPFC activation level (*p* = 0.53) or up-regulation in dlPFC during regulate trials (*p* = 0.52).

### Physiological data

The physiological data analysis showed no significant difference between groups (breathing: *p* = 0.36; pulse: *p* = 0.45), and no differences in physiology during *regulation* in comparison to *watch* trials (breathing: *p* = 0.26; pulse: *p* = 0.36; group task interaction: breathing: *p* = 0.56; pulse: *p* = 0.46). Average breathing rate of all participants was 18 breaths/min and average pulse rate was 66 beats/min.

### Training evaluation

Both groups demonstrated high willingness to return for a second session after the training (Table 2). While participants from the neurofeedback group felt slightly less comfortable in the scanner than control participants, this difference was not significant (Table 3). Both groups reported that the reappraisal instruction facilitated anxiety regulation (Table 3). Neurofeedback participants indicated that neurofeedback was useful both in general, as well as specifically for selecting the reappraisal strategy (Table 3). While participants in the control group found focusing on the aesthetics of the spider and humanizing most successful, the neurofeedback participants chose emphasizing the spider's powerlessness and humanizing as the two most powerful reappraisal strategies (Table 4).

**Table 3:** **Training evaluation**.

**Variables (mean ± SD)**	**Control group**	**Neurofeedback group**	***p*-value**
Comfortable in scanner?	5.8 (1.3)	5.0 (1.7)	0.27
Helpfulness reappraisal strategy	5.3 (1.0)	5.1 (0.6)	0.43
Helpfulness neurofeedback		5.4 (1.6)	
Neurofeedback helped select the reappraisal strategy		5.4 (1.1)	

*Participants rated helpfulness of the instruction and neurofeedback, and how comfortable they were in the scanner environment (Likert scale 1–7)*.

**Table 4:** **Evaluation reappraisal strategies**.

	**Control group**	**Neurofeedback group**
Spider aesthetics	56% (*n* = 5)	22% (*n* = 2)
Powerlessness of the spider	11% (*n* = 1)	44% (*n* = 4)
Humanizing the spider	44% (*n* = 4)	44% (*n* = 4)
Safe environment	11% (*n* = 1)	11% (*n* = 1)

*Percentage of participants finding a reappraisal strategy successful (“which would you recommend?”), several options could be named*.

### Follow-up assessment of fear

**Figure 6:**
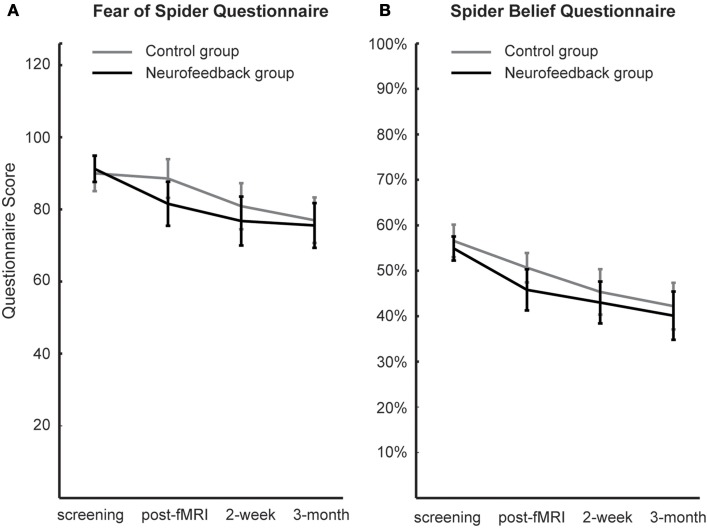
**Spider fear**. Long-term development of spider fear, as assessed with the Fear of Spider Questionnaire **(A)** and the Spider Belief Questionnaire **(B)**, is depicted. While neurofeedback participants demonstrated less anxiety after scanning (post-fMRI), this group difference was not significant and was washed out during the follow-up period (2-week, 3-month).

**Figure 7:**
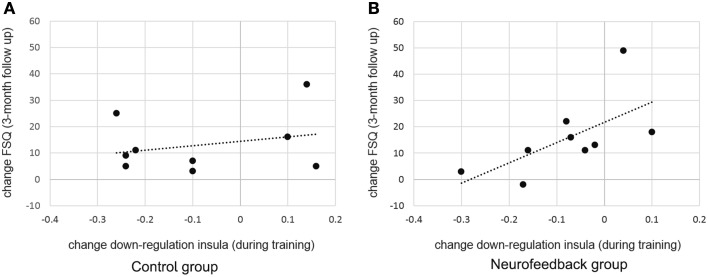
**Predicting long term change in spider fear**. While there was no significant relation between training success and long term fear reduction in the control group **(A)**, individual differences in achieved down-regulation of insula activation level (change from *localization run to last experimental run*) predicted long-term reduction in spider fear (change from *screening* to *3-month follow-up*, Fear of Spider Questionnaire [FSQ] depicted, *r* = 0.64) within the neurofeedback group **(B)**.

When assessed at follow-up, both groups achieved a significant long-term decrease of spider fear, with group differences being attenuated over time [reduction in spider fear: FSQ: *F*_(1, 16)_ = 23.0, *p* < 0.001, η^2^*_p_* = 0.59, SBQ: *F*_(1, 16)_ = 35.1, *p* < 0.001, η^2^*_p_* = 0.690, Figure 6]. Importantly, this long-term reduction in spider fear (*screening* to *3-month follow-up*) correlated with the ability to down-regulate insula activation during neurofeedback training (*localization run to last experimental run*) in neurofeedback participants (FSQ: *r* = 0.64, *p* < 0.05; SBQ: *r* = 0.57, *p* = 0.05, Figure 7) but not in control participants (FSQ: *r* = 0.26, *p* = 0.49; SBQ: *r* = 0.13, *p* = 0.73). Individual differences in efficiency of regulation of brain activation levels therefore predicted change in individual long-term improvement.

## Discussion

We investigated the effect of fMRI neurofeedback training on brain regions involved in fear processing and symptom reduction in patients with spider phobia. Our results demonstrate that neurofeedback participants exhibited lower levels of anxiety than control participants at the end of training. Second, neurofeedback participants, compared to control participants, achieved down-regulation of a region important for anxiety expression (insula), which in turn correlated with improvements in long term anxiety symptoms in these participants.

All participants maintained high prefrontal activation levels during reappraisal, indicating recruitment of regions supporting cognitive reappraisal (Delgado et al., 2008; Ochsner et al., 2012). However, only the neurofeedback group showed a concurrent attenuation of the response in the insula, which grew stronger over time, as expected during successful anxiety regulation (Schienle et al., 2007; Hauner et al., 2012). Decrease of insula activation levels has been shown to be a valid predictor of long term reduction of spider fear (Schienle et al., 2007; Hauner et al., 2012). Neurofeedback participants hence demonstrated the expected modification of brain activation pattern, suggesting the efficiency of cognitive reappraisal strategies for anxiety regulation. Accordingly, achieved attenuation of insula activation levels was accompanied by a reduction of subjective anxiety levels in neurofeedback participants relative to controls. Second, only neurofeedback participants achieved down-regulation of insula activation levels by cognitive reappraisal during regulation in comparison to watch trials. Capacity to down-regulate has been linked to safety learning and successful regulation in healthy subjects (Delgado et al., 2008; Pollak et al., 2010). Group differences in achieved down-regulation of insula activation levels were not reflected in subjective anxiety ratings, nor physiological control data. A possible explanation is that subjective ratings measured on a five-point Likert scale, as well as heart and breathing rate measured during scanning may not be sensitive enough indicators for capturing subtle differences in regulation success. It has previously been shown that heart rate is not a sensitive measure of anxiety regulation even in much larger samples (Aldao and Mennin, 2012; Cristea et al., 2014), and breathing rate is generally not strongly correlated with anxiety levels (Prigatano and Johnson, 1974; Sarlo et al., 2002). Importantly however, observed individual differences in down-regulation of insula activation levels were predictive of long-term changes in fear. While a sustained group difference in fear could not be shown, individually achieved down-regulation of insula predicted fear reduction 3 months after the training. This demonstrates that achieved self-regulation of insula during training was indeed relevant for later behavioral improvement.

Generally, the presented findings add to accumulating evidence that regional changes in brain activation levels can be a valid indicator of therapeutic change (Schienle et al., 2007; Goldin et al., 2009; New et al., 2009; Hauner et al., 2012; Manber-Ball et al., 2013). Observed group differences could not be attributed to differences in engagement or compliance. Participants showed similar baseline levels of subjective anxiety, right insula response, and left dlPFC response, as well as baseline ability to regulate anxiety. Also, both groups reported equal levels of motivation, and expectation of success prior to the training, reported a high level of comfort in the scanner, high helpfulness of the reappraisal instruction, and indicated a comparable desire to return for future sessions.

fMRI neurofeedback training has been previously conceptualized as a method that combines principles of cognitive-behavioral therapy with brain stimulation approaches (Linden et al., 2012). Within this framework, the advantage of neurofeedback training in comparison to physical brain stimulation is that voluntary self-regulation is a self-controlled process, and therefore more accessible in the long run. The assumed mechanism in neurofeedback training is learning. Feedback is expected to facilitate learning through at least two mechanisms: “explicit representational learning” of the strategy and “implicit reinforcement learning” after successful trials (Goebel et al., 2010; Weiskopf, 2012; Sulzer et al., 2013). Additionally, it has been suggested that learning during neurofeedback training may be enhanced by increasing the individuals' self-efficacy (Sarkheil et al., 2015). The presented data support a facilitating effect of neurofeedback for learning of fear regulation, as group differences emerged gradually and became more pronounced over time. The current study therefore corroborates previous studies showing that healthy participants can learn to self-regulate activation levels in various brain regions (Caria et al., 2012; Weiskopf, 2012), including the insula (Caria et al., 2007, 2010). The presented results also show for the first time that patients with high levels of anxiety can achieve self-regulation of insula activation levels when guided by feedback. Furthermore, our data supports previous research demonstrating that cognitive strategies can be successfully shaped by neural feedback, leading to symptom reduction in chronic pain patients (DeCharms et al., 2005), depressed patients (Linden et al., 2012), and participants with subclinical levels of anxiety (Scheinost et al., 2013). A previous study with patients with subclinical levels of contamination anxiety provided participants with feedback on activation levels of a brain region implicated in anxiety provocation (orbitofrontal cortex) during anxiety regulation. The neurofeedback group achieved a sustained reduction of anxiety in comparison to a sham feedback control group. The presented data further substantiate these results, showing that neurofeedback can enhance learning of anxiety regulation.

In the current study feedback was presented intermittent, using a novel dual feedback display. Intermittent feedback paradigms have been previously applied in healthy participants, but not in patients (Bray et al., 2007; Johnson et al., 2012; Stoeckel et al., 2014). The rationale for presenting dual intermittent neurofeedback was to provide patients with a richer representation of their current brain processing than possible with single region neurofeedback. Different to newly emerging methods for network-based connectivity neurofeedback, which capture the interaction between brain regions (Ruiz et al., 2014; Zilverstand et al., 2014), dual neurofeedback is not a direct measure of brain processing between two select brain regions. It is however a method of maximizing relevant information content of the feedback signal, as it allows to simultaneously target several aspects of a complex behavior through training. While a dual neurofeedback display may be challenging for certain groups of patients, the participants in the presented study reported that the feedback provided was helpful in selecting the reappraisal strategy. The presented results show that the approach is feasible, and may be used in clinical populations. While the current results confirm that behavioral effects can be achieved within a single session of neurofeedback training (Sulzer et al., 2013), patients groups with more severe anxiety disorders may benefit from receiving multiple sessions of training (Scheinost et al., 2013).

A limitation of the current study is the modest sample size. To increase homogeneity of the sample only females with spider phobia were recruited, and the generalization of the results to males remains to be determined. The lack of a sham feedback group may also be seen as a limitation. However, previous research found that sham feedback may induce a negative performance bias, which can limit performance of the control group (Johnson et al., 2012; Stoeckel et al., 2014). A non-neurofeedback control group with blinding of participants therefore seemed the strictest design choice available. The presented data confirmed that motivation and expectation effects were well controlled for.

In summary, the conducted study provides first evidence that dual intermittent neurofeedback has a facilitating effect on anxiety regulation in spider phobia. Our results support the idea that self-supervising anxiety regulation by neurofeedback is feasible and can facilitate anxiety regulation. We therefore suggest that neurofeedback training may be incorporated as a therapeutic tool in future clinical trials. Because of common cognitive-behavioral trajectories and neurophysiological pathways, we believe that the presented approach could be extended to a broader range of anxiety disorders.

### Conflict of interest statement

The authors declare that the research was conducted in the absence of any commercial or financial relationships that could be construed as a potential conflict of interest.
