# Supplementary material for: fMRI neurofeedback facilitates anxiety regulation in females with spider phobia
Source: Front Behav Neurosci. 2015 Jun 8;9:148. doi: 10.3389/fnbeh.2015.00148 (PMC4458693; doi:10.3389/fnbeh.2015.00148)
Supplement: Supplementary file 1 [file DataSheet1.PDF]

Supplementary Material

fMRI neurofeedback facilitates anxiety regulation in females with spider phobia

Anna Zilverstand[1,2]*, Bettina Sorger[1], Pegah Sarkheil[1,3], Rainer Goebel[1,4]

[1]Department of Cognitive Neuroscience, Maastricht University, Maastricht, Netherlands
[2]Department of Psychiatry, Icahn School of Medicine at Mount Sinai, New York, NY, USA
[3]Department of Psychiatry, Psychotherapy and Psychosomatics, RWTH Aachen University Hospital, Aachen, Germany
[4]Department of Neuroimaging and Neuromodeling, Netherlands Institute for Neuroscience, Amsterdam, Netherlands

*** Correspondence:** Anna Zilverstand, Department of Cognitive Neuroscience, Maastricht University, Maastricht, Netherlands, Oxfordlaan 55, 6229 EV Maastricht, Netherlands.
anna.zilverstand@gmail.com

1. Supplementary Figures and Tables

1.1. Supplementary Tables

Supplementary Table 1. Target Regions. The coordinates and size (in functional voxels 3x3x3 mm3) of the individually localized target regions in the left dlPFC and right insula are reported for all subjects. Group average coordinates came close to the target coordinates, and the size of the defined regions (e.g., number of voxels) was comparable across groups.

Supplementary Table 1 A. Talairach coordinates of left dlPFC target regions.

**Control group**					**Neurofeedback group**				
	**x**	**y**	**z**	**Number of** functional voxels (3x3x3 mm^3)		**x**	**y**	**z**	**Number of** functional voxels (3x3x3 mm^3)
S01	-32	35	33	21	S10	-46	26	26	12
S02	-47	16	28	17	S11	-48	23	39	10
S03	-37	33	20	19	S12	-41	36	26	24
S04	-48	27	33	6	S13	-42	30	24	6
S05	-26	41	40	11	S14	-36	36	19	11
S06	-33	35	30	11	S15	-33	45	28	12
S07	-38	36	17	8	S16	-36	27	39	13
S08	-44	24	29	25	S17	-47	16	34	24
S09	-34	39	30	11	S18	-32	44	34	16
**mean**	**-38**	**32**	**29**	**14**	**mean**	**-40**	**31**	**30**	**14**
**SD**	**7**	**8**	**7**	**6**	**SD**	**6**	**10**	**7**	**6**
**target**	**-43**	**28**	**30**		**target**	**-43**	**28**	**30**	

Supplementary Table 1 B. Talairach coordinates of right insula target regions.

Control group					Neurofeedback group				
	**x**	**y**	**z**	**Number of** functional voxels (3x3x3 mm^3)		**x**	**y**	**z**	**Number of** functional voxels (3x3x3 mm^3)
S01	46	3	6	11	S10	47	0	-1	9
S02	37	20	-1	17	S11	37	10	10	10
S03	39	-6	12	4	S12	43	2	2	17
S04	32	13	14	8	S13	36	7	8	23
S05	40	15	5	25	S14	39	1	4	7
S06	37	4	11	11	S15	42	10	5	26
S07	42	10	4	22	S16	33	16	9	26
S08	42	7	10	9	S17	31	13	13	7
S09	42	18	3	6	S18	31	21	12	8
**mean**	**40**	**9**	**7**	**12**	**mean**	**38**	**9**	**7**	**15**
**SD**	**4**	**8**	**5**	**8**	**SD**	**6**	**7**	**5**	**8**
**target**	**37**	**11**	**3**		**target**	**37**	**11**	**3**	

Supplementary Table 2. Group differences in down-regulation. A whole-brain random-effects GLM analysis investigating group differences in down-regulation (reduced activation levels during *regulation* in comparison to *watch* trials) during training was conducted. Results demonstrated a significant group difference with greater down-regulation in the neurofeedback than the control group. This effect was apparent within a right-lateralized network of regions typically activated during anxiety expression. The lateralization of the effect, with a strong dissociation between left and right dlPFC, is in accordance with the previous literature (Etkin and Wager, 2007; Delgado et al., 2008; Ochsner et al., 2012). Within the right insula the focus of the effect (x/y/z = 32/22/9) was in the vicinity of the coordinates for defining the neurofeedback target regions (x/y/z = 37/11/3).

**Regions showing an increased difference in the neurofeedback group**			
		Brodmann's Areas	x/y/z (no of voxels)
Anterior cingulate cortex	L	24, 32	-2/31/35 (80)
	R	24, 32	2/29/33 (44)
Dorsolateral prefrontal cortex	L		---
	R	9	49/13/25 (223)
Ventrolateral prefrontal cortex	L		---
	R	44,47	48/7/14 (37)
Insula	L		---
	R	13	32/22/9 (203)
**Regions showing a reduced difference in the neurofeedback group**			

L = Left Hemisphere, R = Right Hemisphere
Talairach coordinates of the most significant voxel and the number of significantly activated functional voxels (3x3x3 mm^3) are reported.

1.2. Supplementary Figures

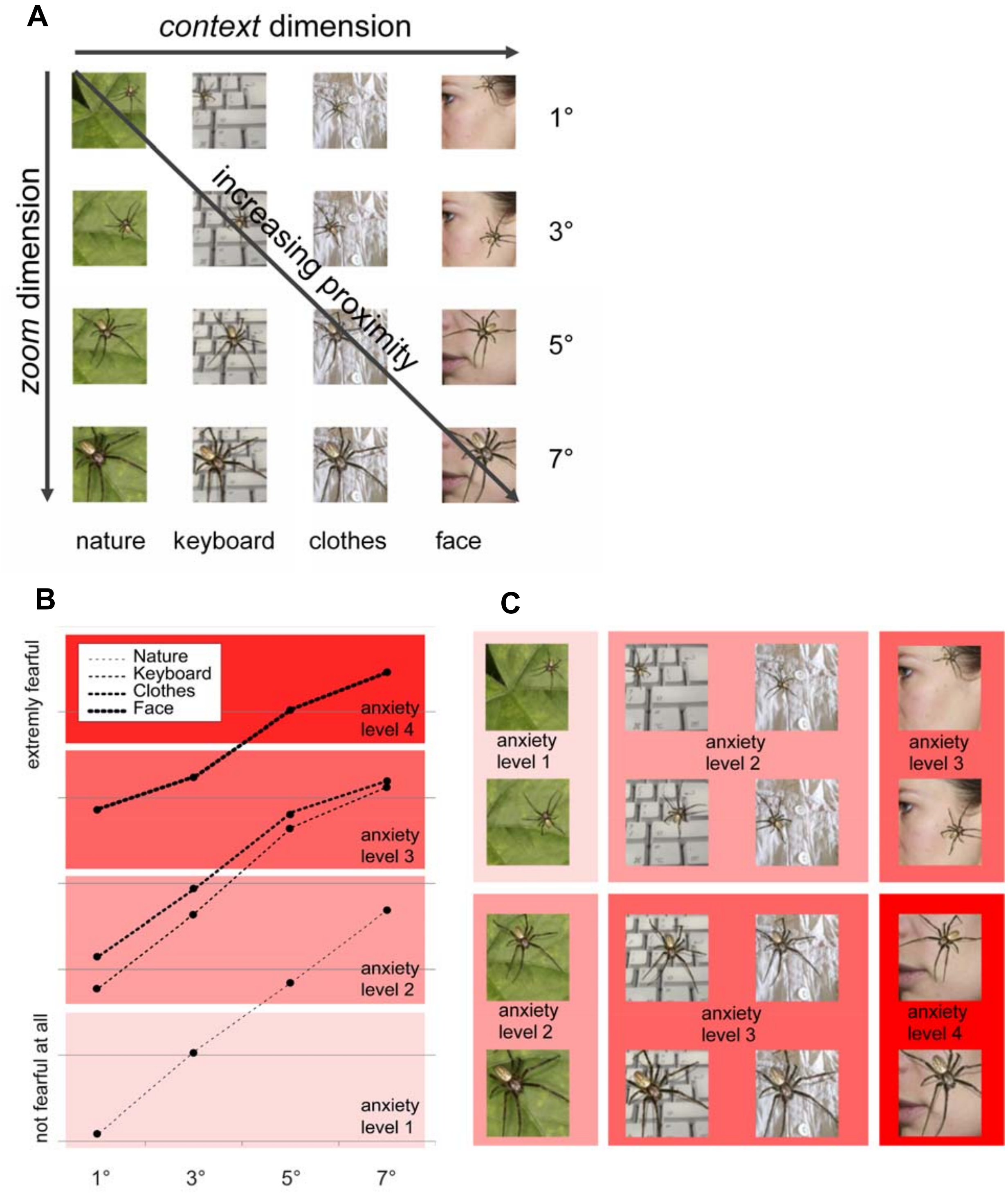

Supplementary Figure 1. Stimuli.

A parametric spider picture set was created through systematic manipulation of the spider's context, size and posture (**A**). Spiders were mounted on four different sorts of backgrounds. Proximity in context increased across backgrounds (context: nature > keyboard > clothes > face). Along the zoom dimension the spider increased in size (1, 3, 5, or 7 degree visual angle) and became more oriented towards the viewer. Both stimulus manipulations were hypothesized to gradually intensify provoked anxiety in spider phobics. A total of 48 stimuli were created for the behavioral pilot study. Twenty spider females (age = 22.6 years) with high spider fear (average SPQ score = 20.3) [Spider Phobia Questionnaire (SPQ) Score ≥ 14, (Klorman et al., 1974)] rated the stimuli. They were asked to indicate "their initial reaction" to the randomly presented pictures on a digital visual analogue scale anchored with 'not fearful at all' and 'extremely fearful' (**B**). Statistical analysis showed that as hypothesized anxiety increased linearly along both dimensions (context dimension: $\eta p2 = 0.66$, significant in 19/20 participants and group level; zoom dimension: $\eta p2 = 0.73$, significant in 19/20 participants and group level) (Zilverstand et al., 2013). By dividing the covered range into four levels (**B**), the sixteen stimulus subcategories were grouped into four different stimulus categories according to provoked anxiety level (anxiety level 1, anxiety level 2, anxiety level 3, anxiety level 4), as depicted by the background colors (**C**). For the current study eight stimuli from each of the four levels were selected.

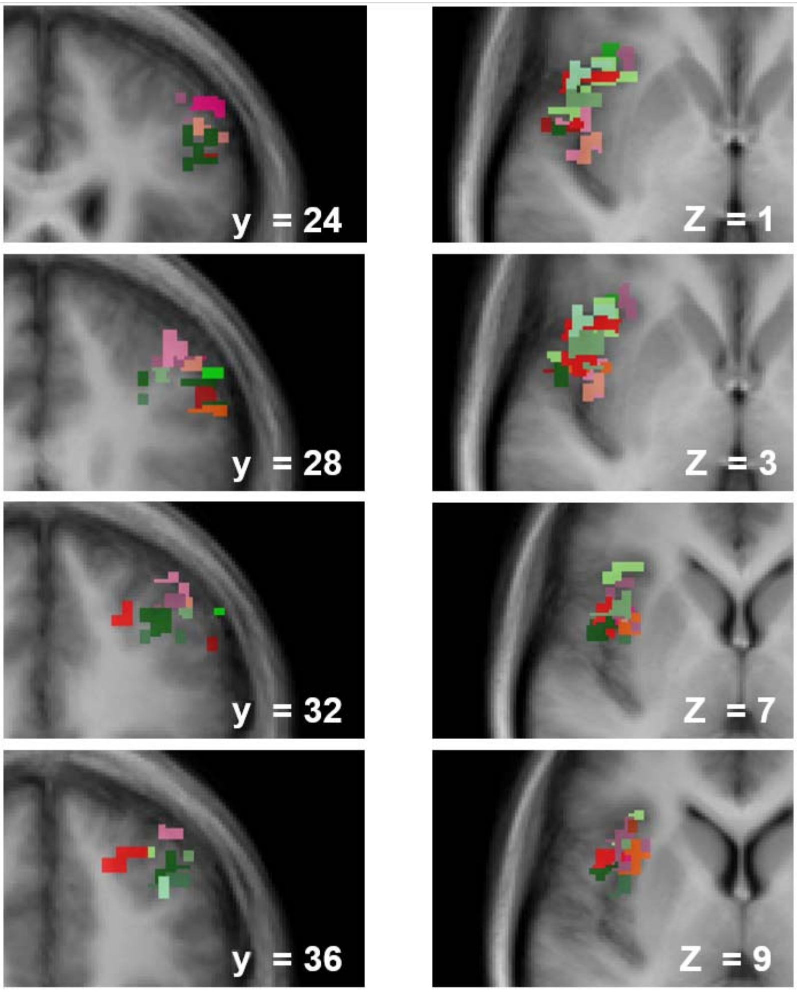

Supplementary Figure 2. Localization of target regions.
The individual left dlPFC (**A**) and right insula (**B**) target regions are depicted for neurofeedback (red tints) and control participants (green tints). All regions were defined based on the localization run. The left dlPFC target regions were delineated by contrasting *regulate trials* versus *resting*, and selecting the closest cluster around the target coordinates x = -43, y = 28, z = 30. The right insula target regions were defined by contrasting *watch trials* versus *resting* and using the target coordinates x = 37, y = 11, z = 3. As depicted, all target regions were confined within insula and dlPFC and showed strong spatial overlap across groups.

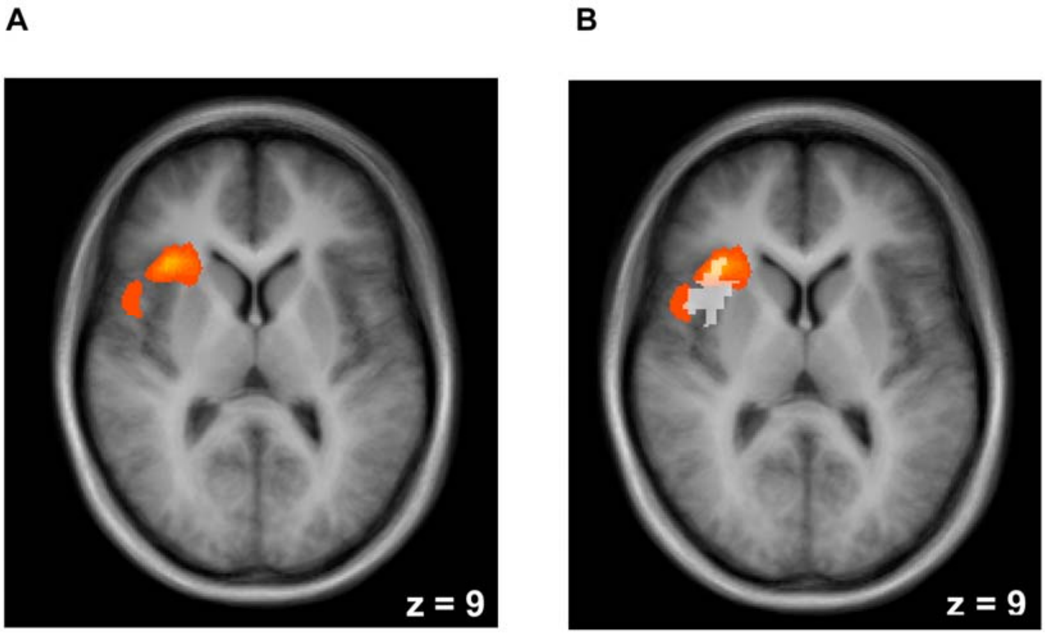

Supplementary Figure 3. Whole brain analysis for down-regulatory effect.
The whole-brain random-effects GLM analysis of the down-regulatory effect during experimental runs demonstrated a significant group difference in the insula ($p<0.05$, corrected at cluster level, see ST 2), with a stronger down-regulation effect (reduced response during regulation in comparison to watch trials) in neurofeedback participants (A). Within the right insula the focus of this effect (x/y/z = 32/22/9) was in the vicinity of the average coordinate of the defined target regions (x/y/z = 37/11/3), as indicated by overlaying the combined individual ROIs on the exemplary slice (B).

2. References

Delgado, M. R., Nearing, K. I., Ledoux, J. E., and Phelps, E. a (2008). Neural circuitry underlying the regulation of conditioned fear and its relation to extinction. *Neuron* 59, 829–38. doi:10.1016/j.neuron.2008.06.029.

Etkin, A., and Wager, T. D. (2007). Functional neuroimaging of anxiety: a meta-analysis of emotional processing in PTSD, social anxiety disorder, and specific phobia. *Am. J. Psychiatry* 164, 1476–1488. doi:10.1176/appi.ajp.2007.07030504.

Klorman, R., Weerts, T. C., Hastings, J. E., Melamed, B. G., and Lang, P. J. (1974). Psychometric description of some specific-fear questionnaires. *Behav. Ther.* 5, 401–409. doi:10.1016/S0005-7894(74)80008-0.

Ochsner, K. N., Silvers, J. a, and Buhle, J. T. (2012). Functional imaging studies of emotion regulation: a synthetic review and evolving model of the cognitive control of emotion. *Ann. N. Y. Acad. Sci.* 1251, E1–24. doi:10.1111/j.1749-6632.2012.06751.x.

Zilverstand, A., Sorger, B., Kaemingk, A., and Goebel, R. *Towards therapy in the MRI scanner : cognitive reappraisal of parametrically modulated anxiety in spider phobia*. 43rd annual meeting of the Society for Neuroscience; 2013 Nov 9-13; San Diego, CA, USA.
